# The Suppression of Maternal–Fetal Leukemia Inhibitory Factor Signal Relay Pathway by Maternal Immune Activation Impairs Brain Development in Mice

**DOI:** 10.1371/journal.pone.0129011

**Published:** 2015-06-04

**Authors:** Tsuyoshi Tsukada, Eriko Simamura, Hiroki Shimada, Takuma Arai, Nobuaki Higashi, Takuya Akai, Hideaki Iizuka, Toshihisa Hatta

**Affiliations:** 1 Department of Neurosurgery, Kanazawa Medical University, Uchinada, Ishikawa 920–0293, Japan; 2 Department of Anatomy, Kanazawa Medical University, Uchinada, Ishikawa 920–0293, Japan; Virgen Macarena University Hospital, School of Medicine, University of Seville, SPAIN

## Abstract

Recent studies in rodents suggest that maternal immune activation (MIA) by viral infection is associated with schizophrenia and autism in offspring. Although maternal IL-6 is though t to be a possible mediator relating MIA induced these neuropsychiatric disorders, the mechanism remains to be elucidated. Previously, we reported that the maternal leukemia inhibitory factor (LIF)–placental ACTH–fetal LIF signaling relay pathway (maternal–fetal LIF signal relay) promotes neurogenesis of fetal cerebrum in rats. Here we report that the maternal–fetal LIF signal relay in mice is suppressed by injection of polyriboinosinic-polyribocytidylic acid into dams, which induces MIA at 12.5 days post-coitum. Maternal IL-6 levels and gene expression of placental *suppressor of cytokine signaling* 3 (*Socs3*) increased according to the severity of MIA and gene expression of placental *Socs3* correlated with maternal IL-6 levels. Furthermore, we show that MIA causes reduction of LIF level in the fetal cerebrospinal fluid, resulting in the decreased neurogenesis in the cerebrum. These findings suggest that maternal IL-6 interferes the maternal–fetal LIF signal relay by inducing SOCS3 in the placenta and leads to decreased neurogenesis.

## Introduction

Epidemiological studies show that maternal viral infection during pregnancy increases the risk of schizophrenia and autism in the offspring [1–4]. In recent years, maternal immune activation (MIA) in rodents due to viral infection, not direct infection of the fetuses, is the main cause of schizophrenia and autism developed in the offspring [5]. Injection of polyriboinosinic-polyribocytidylic acid [poly (I:C)], a synthetic analog of double-stranded RNA, causes MIA and is used in rodents to mimic maternal viral infection. The injection induces a so-called cytokine storm: an increase in pro-inflammatory cytokine levels such as interleukin-6 (IL-6), tumour necrosis factor-α and interleukin-1β [6, 7]. Among the pro-inflammatory cytokines induced by poly (I:C), IL-6 from the mouse maternal serum (MS) was identified as a likely mediator able to impair fetal brain development [8–10]. Although both direct and indirect effects of IL-6 on fetal brain development are suggested, underlying mechanisms remain to be elucidated.

Here we propose a mechanism, by which MIA suppresses fetal brain development. We have previously shown that one of the pro-inflammatory cytokine belonging to the IL-6 family cytokines, leukemia inhibitory factor (LIF), is required for fetal brain development [11, 12]. We also demonstrated that rat maternal LIF induces adrenocorticotropic hormone (ACTH) from the placenta, which in turn stimulates fetal nucleated red blood cells to secrete LIF, leading finally to neurogenesis in the cerebrum of the fetus [13, 14]. This maternal—fetal signal relay pathway of maternal LIF–ACTH–fetal LIF via the placenta (maternal–fetal LIF signal relay) was shown in rats at mid-gestational stage [14]. We hypothesized that an excess of IL-6 in the maternal serum induced by MIA interferes with the maternal–fetal LIF signal relay due to the shared signal transducer gp130 [15]. In this study, we examined the effects of MIA on the expression of molecules involved in the maternal–fetal LIF signal relay in the placenta and fetal brain. We then demonstrated the relation of placental suppressor of cytokine signaling 3 (SOCS3), which negatively regulate gp130 mediated signalling by inhibiting the phosphorylation of STAT3 [16] and maternal IL-6 in MIA.

## Materials and Methods

### Animals

Female C57BL/6J mice aged 8–24-weeks-old were used in this study. The mice were maintained under standard laboratory conditions. Food and water were available *ad libitum*. A female mouse was housed with a male mouse overnight, and the day when a vaginal plug was found in the morning was designated as 0.5 days post-coitum (0.5 dpc). Dams were anaesthetized by intraperitoneal (i.p.) injection of pentobarbital (30 mg/kg), and embryos were dissected with the placenta. Embryos were scored for crown-rump length (CRL) with ImageJ software [17] and used in the following studies when the CRL was in the predefined CRL range. The CRLs used in this study are summarized in S1 Fig. There were no significant differences at 12.5 dpc and 14.5 dpc among the groups. All procedures in this study were performed in strict accordance with the guidelines for the Care and Use of Laboratory Animals of Kanazawa Medical University. The protocol was approved by the Committee on the Ethics of Animal Experiments of the Kanazawa Medical University (Permit Number: 2012–25, 2013–16, 2014–2, 2014–33). All surgery was performed under sodium pentobarbital anesthesia, and all efforts were made to minimize suffering.

### Sampling of fetal fluids

After embryo were dissected out and scored for CRL, we collected blood and CSF in turn using micro pipettes under microscope (Leica Microsystems)[12]. o keep the purity of the obtained samples, we pay maximum attention not to be contaminated with amniotic fluid and/or fetal blood. That is, in collecting fetal blood from fetal hearts, we pay attention not to aspirate amniotic fluid by keeping fetus semi dry condition. In collecting fetal CSF, we pay attention not to aspirate amniotic fluid and fetal blood. We first examined the purity of obtained CSF not to be contaminated by fetal blood in preliminary study (3 fetuse). The purity of obtained CSF was 99.99%. Fluid samples of fetuses derived from one dam were mixed together as the pooled sample for the litter, the pooled sample approximately amount to 20 μl. Fetuses were used not only for fluid sample, but also for histological study, we obtained fetal serum and fetal CSF 5μl per fetus. Sampling was done without regard to sex of fetuses.

### MIA and LIF injection

At 12.5 dpc, dams were administered an i.p. injection of poly (I:C) (Sigma Aldrich, St. Louis, MO) at 4 mg/kg or 20 mg/kg based on the weight of the poly (I:C) itself [8]. A model of neurodevelopmental disorder using MIA resulted in severe MIA equivalent to fulminant infection in human [8, 10, 18–20]. In clinical settings, all the infected mothers would not suffer from fulminant infection. Therefore, we also examined the effects of comparatively low dose of poly (I:C). In this study, the groups injected with poly (I:C) at 4 mg/kg or 20 mg/kg are presented as Poly 4 and Poly 20, respectively. We injected intraperitoneally mouse recombinant LIF (rLIF; Millipore, Billerica, MA) into dams at 5 μg/kg body weight [14] at 12.5 or 13.5 dpc. The injection volume was unified into 0.01 ml/g. Control was injected with 0.01 ml/g volume of only saline.

### Sandwich ELISA

At 24 h after the injection of poly (I:C) or saline at 12.5 dpc, maternal serum (MS), fetal serum (FS) and fetal cerebrospinal fluid (CSF) were isolated, mixed with an equal volume of 50% ethylene glycol respectively and stored at −30°C. Samplings were also performed at 3 h after the injection of LIF or saline at 13.5 dpc as mentioned above. ACTH was detected using mouse anti-ACTH monoclonal antibody (mAb; Santa Cruz Biotechnology, Santa Cruz, CA) for capture and rabbit anti-ACTH polyclonal antibody (pAb; Chemicon, Temecula, CA) labelled with biotin for detection [14]. The ACTH assay was validated (n = 3) with the following results: Sensitivity was 0.14 nM, intraassay coefficient of variance was 10.2% and interassay coefficient of variance was 20.1%. LIF was detected using a sandwich ELISA, in which pAb (1:500; no. L9152; Sigma Aldrich) was used as the capture antibody and biotinylated mouse anti-LIF pAb (1:250; BAF449; R&D Systems, Minneapolis, MN) as the detection antibody. The LIF assay was validated (n = 3) with the following results: Sensitivity was 37.0 pg/ml, intraassay coefficient of variance was 8.5% and interassay coefficient of variance was 12.7%. IL-6 was detected using sandwich ELISA kits (RayBio Technology, Inc., Norcross, GA) following the manufacturer’s instructions. All plates were treated with Starting Block blocking buffer (Pierce, Rockford, IL), and biotin was revealed with ExtrAvidin–peroxidase conjugate (Sigma Aldrich). Horseradish peroxidase (HRP) activity was detected with 1-Step Ultra TMB Substrates (Pierce). Samples were measured in triplicate by 2104 En Vision (PerkinElmer, Waltham, MA). Three or four dams were used for each group.

### Quantitative real-time PCR

Placentas were quickly dissected after the isolation of the serum and CSF of fetuses at 12.5 dpc and 13.5 dpc. Total RNA of placentas was extracted using RNeasy (Qiagen, Valencia, CA). Expression levels of *Proopiomelanocortin* (*Pomc*), *Il-6st* (*gp 130*), *Lifr* (*Lif receptor*), *Il-6r* (*Il-6 receptor*) and *Socs3* were examined using a gene expression assay (Applied Biosystems, Foster City, CA). POMC is the precursor of ACTH and melanocortin peptides. A total of 2 μg of RNA was reverse-transcribed using SuperScript III reverse transcriptase (Invitrogen, Carlsbad, CA). Quantitative PCR was performed using cDNA with TaqMan gene expression master mix (TakaRa Bio, Shiga, Japan), and TaqMan 18S ribosomal RNA (Applied Biosystems) as an internal control. Reactions were performed in triplicate using ABI Prism 7900HT (Applied Biosystems) and quantified using the delta–delta-cycle threshold (Ct) method (2 ^-ΔΔCt^) [21]. Three or four dams for each group were used, and two fetuses were collected as a pooled sample from each litter.

### Western Blot Analysis

Dissected placentas were quickly stored at −80°C. Western blotting was performed as follows. In brief, placenta from each group was lysed with lysis buffer [1% SDS, 1 mM sodium orthovanadate, Tris (pH 7.4)]. After microwave treatment for 10 s, the lysate was centrifuged at 16,000 ×*g* for 5 min at 15°C to remove insoluble material. Protein contents in obtained samples were determined using the Micro BCA protein assay kit (Pierce). Samples were boiled for 10 min in sample buffer (Wako Pure Chemical Industries, Osaka, Japan). Proteins were then separated by SDS-PAGE and electroblotted onto a PVDF membrane using the iBlot Device (Invitrogen). The membrane was incubated with the indicated antibody after blocking in the Starting Block blocking buffer (Pierce). For primary antibodies, rabbit anti-pSTAT3 mAb (Abcam, Cambridge, MA) and mouse anti-STAT3 mAb (Abcam) were used. The membrane was washed using Tris-buffered saline with Tween 20 and incubated at room temperature with horseradish peroxidase-conjugated anti rabbit or anti mouse secondary antibody. The signal was visualized by enhanced chemiluminescence (Pierce). To reblot the membrane with either primary antibody, the membrane was stripped by Restore Plus Western Blot Stripping Buffer (Thermo Scientific). The band intensities from each blot membrane were quantified using the ImageJ software, and the relative expression was evaluated. Experiments were repeated three times.

### Estimation of volume and total cell number of the cerebral cortex

All measurements were performed on three fetuses from two litters in each group at 18.5 dpc following MIA at 12.5 dpc. Fetuses were fixed in a mixture of 70% methanol and 10% formalin by immersion at room temperature for 24 h and were subsequently embedded in paraffin. Fifteen-μm thick coronal serial sections of the brain were cut and stained with haematoxylin–eosin. From the most anterior portion of the cerebral hemisphere, excluding the rhinencephalon to the most posterior portion, we estimated the volume and total cell number of the right cerebral cortex using a nonbiased stereological method (Stereo Investigator, version 10; Micro BrightField, Williston, VT) and a Leica Imager M1 light microscope. The right cerebral cortex of the hemisphere was outlined following predetermined regions of interest at 10× magnification. The borders of the cerebral cortex were determined based on histological findings as follows: anterior, the rhinencephalon; inferior, the intermediate cortical layer; lateral, the insular cortex and medial, the cingulate cortex (these cortical areas were excluded in this analysis). The Stereo Investigator placed a random point-grid over every 10th section; volume and total cell numbers of the cerebral cortex were estimated using the Cavalieri point counting method and the optical fractionator probe, respectively [22–24]. Nuclei stained with haematoxylin were counted at 40 × oil emersion magnification. The stereological parameters used to estimate the total cell number of the cerebral cortex are summarized in S1 Table. A total of 9 fetuses were tested (control, n = 3 [2 litters]; poly (I:C) 4 mg/kg, n = 3 [2 litters] and poly (I:C) 20 mg/kg, n = 3 [2 litters]).

### EdU-labelling index in the fetal cerebral cortex

To label proliferating neural stem/progenitor cells within the ventricular (VZ)/subventricular zone (SVZ) at 14.5 dpc following MIA at 12.5 dpc, pregnant mice were i.p. injected with 20 mg/kg body weight of 5-ethynyl-2′-deoxyuridine (EdU) 2 h before sacrificing, and the cell’s ability to proliferate was measured using the Click-it EdU Imaging Kits (Invitrogen). Fetuses were fixed in a mixture of 70% methanol and 10% formalin by immersion at room temperature overnight and were subsequently embedded in paraffin. Fifteen-μm thick coronal serial sections from the right cerebral cortex were reacted with Click-it EdU Imaging Kits and were counter stained by Hoechst 3334. The sections for stereological investigation were determined by the following method. The first section, in which an intraventricular foramen was present, was designated as the anterior part of the fetal brain; from there the three consecutive 10th sections along the caudal-to-rostral direction were examined as anterior to the frontal part of the fetal brain. Histological analysis of cortical plate in gp130 deficient fetuses showed decreased cells number compared to controls and did not reveal any differences in anterior, intermediate, and posterior part of cortex [11]. As an intraventricular foramen is easily recognized, we select anterior part of the fetal brain as region of interest for cell counting. EdU-positive and negative cells per section were determined by the optical fractionator method [23] assisted by Stereo Investigator. Cell nuclei were counted in three-dimensional counting frames (30 × 30 × 30 μm^3^) at 40 × oil emersion magnification. The total number of counting frames, automatically generated by Stereo Investigator, was approximately 10 per section. The EdU-labelling index per section was calculated, and the mean value of three sections per foetus was used for further statistical analysis. Four fetuses for each group were used [n = 4 (4 litters)].

### Statistical analysis

All analyses were performed with Statview software. Data in this study are presented as the mean ± SEM. Unless noted otherwise, student's *t*- test was used for comparisons of groups and Pearson's correlation coefficient test was used for correlations analysis and p < 0.05 was accepted as statistically significant. Kruskal–Wallis test was used for analysis of maternal serum and p < 0.05 was accepted as statistically significant.

## Results

### LIF–ACTH–LIF signaling relay pathway in mice

**Fig 1:**
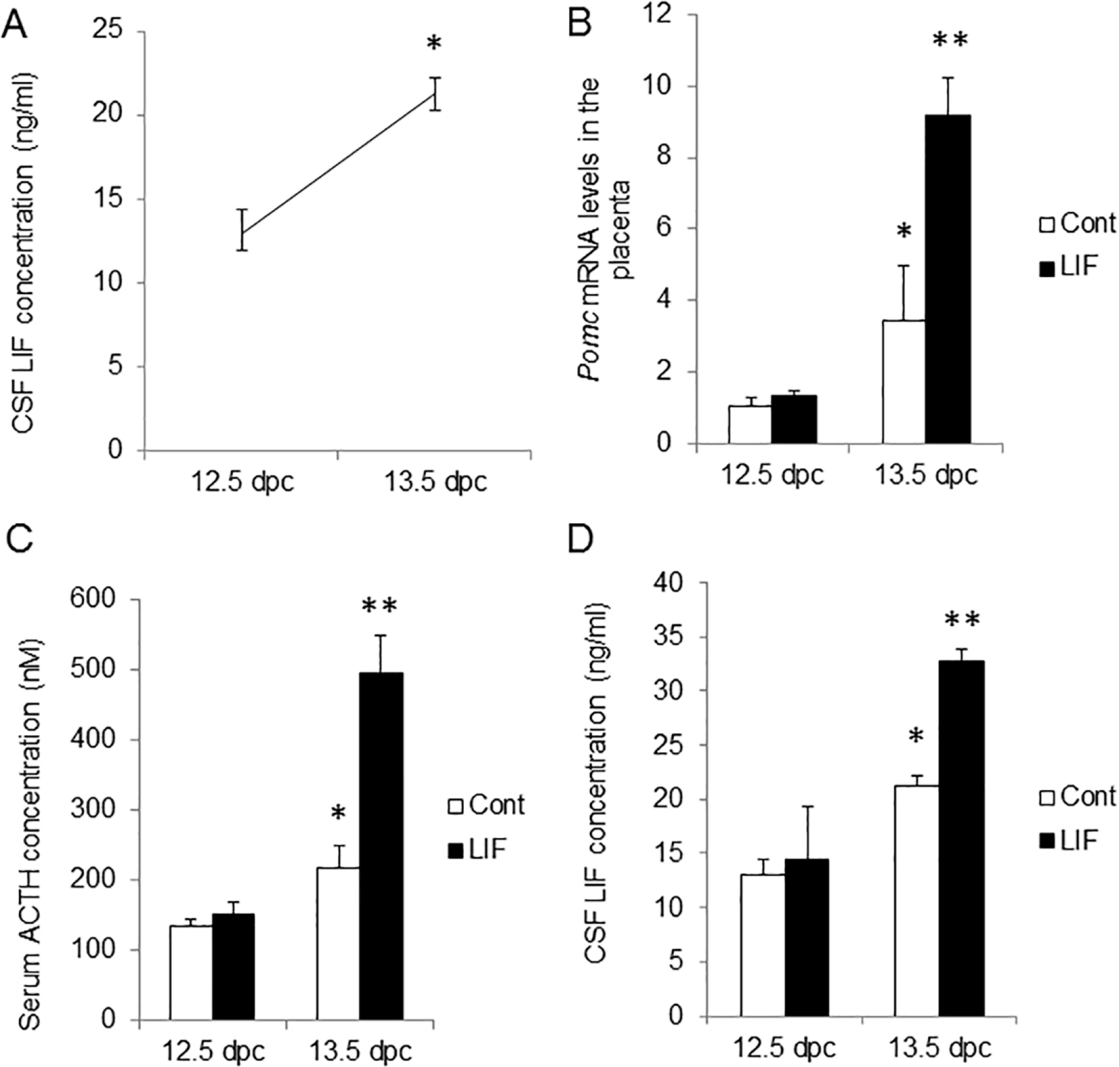
Response of leukemia inhibitory factor (LIF)–adrenocorticotropic hormone (ACTH)–LIF signaling to maternal LIF stimulation in mice. (A) Chronological change of LIF levels in fetal cerebrospinal fluid (CSF). (B) *Pomc* expression levels in the placenta. (C) ACTH level in fetal serum. (D) LIF level in fetal CSF. Reactivity to maternal LIF stimulation in placenta and fetus were examined at 3 h after the injection at 12.5 and 13.5 days post-coitum (dpc). Mouse recombinanat LIF (rLIF) was administered to dams at 5 μg/kg body weight. *, *p* < 0.05 vs. control at 12.5 dpc. **, *p* < 0.05 versus control at 13.5 dpc. Number of dams = 3 or 4 in each group. *Error bars*, SEM.

We have previously shown the existence of a LIF–ACTH–LIF signaling relay pathway via the placenta in rats at 15.5 dpc [14]. In the present study, we first demonstrated that in mice, a similar maternal–fetal LIF signal relay exists by showing the reactivity of placenta and fetuses after exogenous LIF injection into dams. First, concentration of LIF was significantly higher at 13.5 dpc than 12.5 dpc in fetal CSF (Fig 1A). Second, we injected rLIF into dams at 12.5 dpc and 13.5 dpc and measured the *Pomc* expression level (in the placenta), concentration of ACTH (in FS) and concentration of LIF (in fetal CSF) at 3 h after the injection. At 12.5 dpc, neither the placental *Pomc* expression nor the concentration of ACTH and LIF were altered by the exogenous LIF injection. In contrast, at 13.5 dpc, placental *Pomc* expression level and concentration of ACTH in FS significantly increased (Fig 1B and 1C) and concentration of LIF in CSF increased 3 h after LIF injection (Fig 1D). Concentration of ACTH in FS also showed chronological increment (Fig 1C). These results demonstrate that the maternal–fetal LIF signal relay exist in mice as well as in rats.

### Effect of MIA on fetal brain development

**Fig 2:**
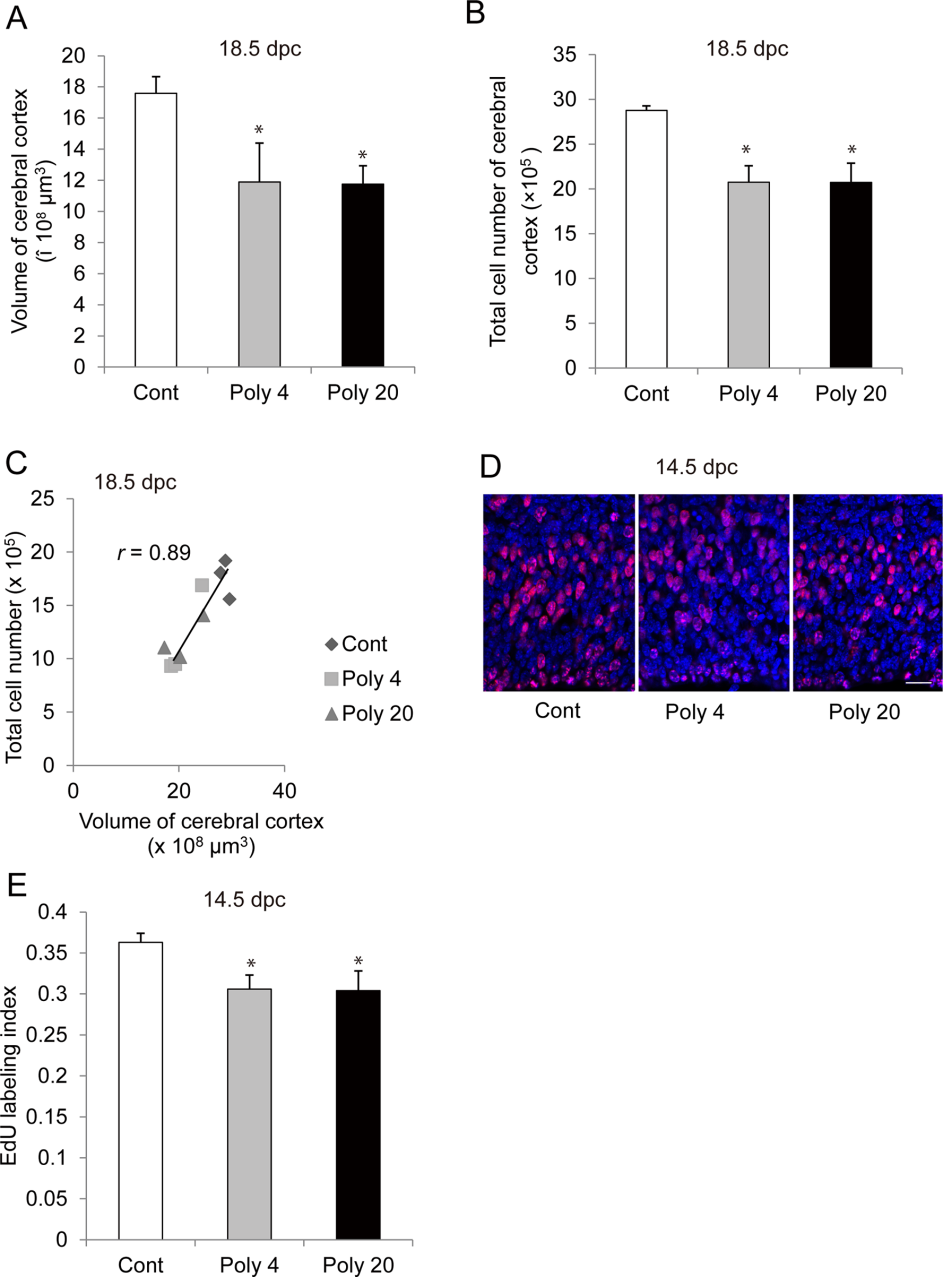
Effect of maternal immune activation (MIA) at 12.5 days post-coitum (dpc) on fetal brain development. (A) Volume of fetal cerebral cortex at 18.5 dpc. (B) Total cell number of cerebral cortex at 18.5 dpc. (C) Correlation between total cell number and volume of fetal cerebral cortex. (D, E) 5-ethynyl-2′-deoxyuridine (EdU) labelling index in the fetal ventricular zone (VZ)/subventricular zone (SVZ) at 14.5 dpc. Cell nuclei were counted at 40 × oil emersion magnification. *Bar*, 20 μm. In polyriboinosinic-polyribocytidylic acid [poly (I:C)] injection groups, fewer EdU-positive cells were observed compared with controls. Cont: control, Poly 4: poly (I:C) 4 mg/kg, Poly 20: poly (I:C) 20 mg/kg. *, *p* < 0.05. For the estimation of volume and total cell number in the cerebral cortex, a total of 9 fetuses were tested (control, n = 3 [2 litters]; poly (I:C) 4 mg/kg, n = 3 [2 litters]; poly (I:C) 20 mg/kg, n = 3 [2 litters]). For EdU immunostaining, n = 4 fetuses from four litters in each group, *Error bars*, SEM.

Based on the current knowledge, MIA affects the fetal brain development. Therefore, we first examined the volume and the total cell number of the dorsolateral cerebral cortex in the right cerebral hemisphere at 18.5 dpc exposed to MIA. Both groups of fetuses from Poly 4 and Poly 20 treated mice had a cerebral cortex with a significantly reduced volume and cell number at 18.5 dpc (Fig 2A and 2B, p < 0.05). The total cell number positively correlated with the volume of the cerebral cortex (Fig 2C). Next, we investigated the effects of MIA on the proliferative activity of neural stem/progenitor cells in the VZ/SVZ, EdU was injected to dams at 14.5 dpc [48 h after poly (I:C) or saline injection, respectively], and embryos were dissected 2 h after injection. Compared with the control group, both Poly 4 and Poly 20 groups displayed a decreased EdU labelling index at 14.5 dpc in favour of a lower proliferation rate (Fig 2D and 2E).

### MIA-induced IL-6 and LIF concentration changes in MS

**Fig 3:**
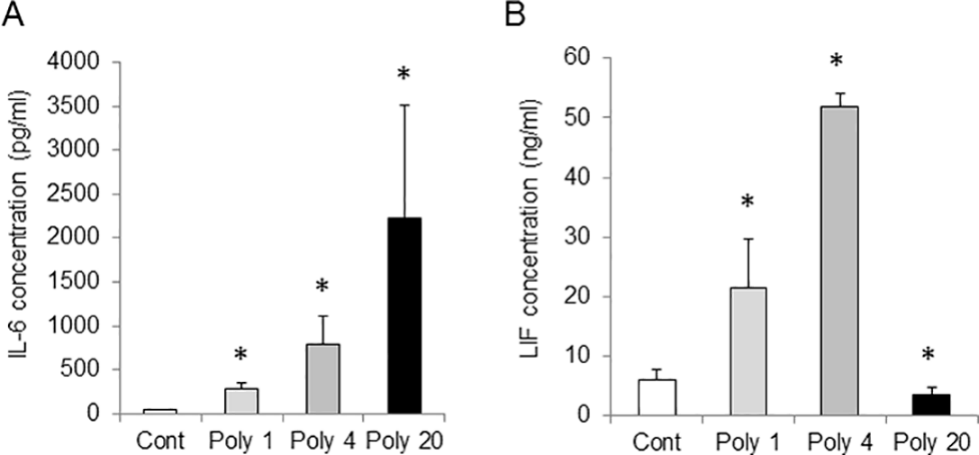
Changes in interleukin-6 (IL-6) and leukemia inhibitory factor (LIF) concentrations in the maternal serum in response to maternal immune activation (MIA). (A) Concentration of IL-6 in maternal serum (MS). (B) Concentration of LIF in MS. MIA was induced by an intraperitoneal (i.p.) injection of 1, 4 or 20 mg/kg polyriboinosinic-polyribocytidylic acid [poly (I:C)] at 12.5 days post-coitum (dpc), maternal serum was analysed 3 h after the injection. Controls were injected with an equal volume of saline (0.01 ml/g body weight). Injection of polyriboinosinic-polyribocytidylic acid [poly (I:C)] at 12.5 dpc induced IL-6 in MS in a dose dependent manner. In contrast, the concentration of LIF in MS was increased by the injection of poly (I:C) at 1 and 4 mg/kg; the concentration of LIF in MS was decreased to the control level by the injection of poly (I:C) at 20 mg/kg. Cont: control, Poly 4: poly (I:C) 4 mg/kg, Poly 20: poly (I:C) 20 mg/kg. *, *p* < 0.05. Number of dams = 3 or 4 in each group. *Error bars*, SEM.

We expected that the dosage of poly (I:C) would have an effect on the concentration of IL-6 and LIF in MS. To clarify the correlation between the severity of MIA induced by poly (I:C) and the concentrations of IL-6 and LIF in MS at 3 h after the poly (I:C) injection, we performed sandwich ELISA. Dams were administered an i.p. injection of poly (I:C) at 1 mg/kg, 4 mg/kg or 20 mg/kg. The concentration of IL-6 in MS increased in a dose-dependent manner of the poly (I:C) injection (Fig 3A, p < 0.05 by the Kruskal–Wallis test). In contrast, compared with the robust increase of the IL-6 level following a higher dosage of poly (I:C), the levels of LIF decreased drastically in MS between Poly 4 and Poly 20 groups (Fig 3B, p < 0.05 by the Kruskal–Wallis test).

### MIA-induced fetal ACTH and LIF level changes

**Fig 4:**
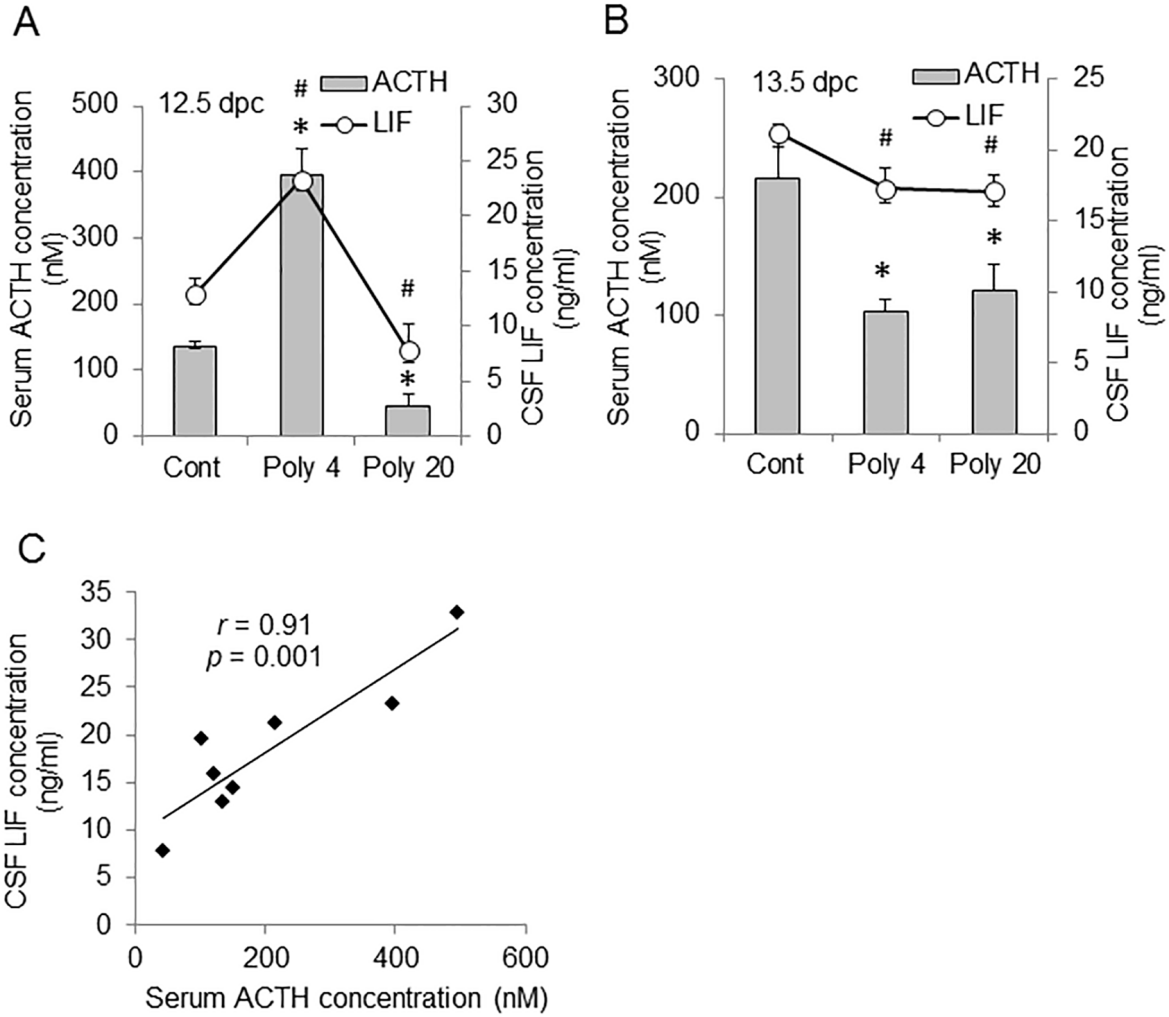
Alteration of fetal adrenocorticotropic hormone (ACTH) and leukemia inhibitory factor (LIF) levels by maternal immune activation (MIA). (A) Concentration of ACTH in fetal serum (FS) and LIF in fetal cerebrospinal fluid (CSF) at 3 h after MIA at 12.5 days post-coitum (dpc). (B) Concentration of ACTH in FS and LIF in fetal CSF at 13.5 dpc. (C) Correlation of LIF level in CSF and ACTH level in FS. MIA was induced by an intraperitoneal (i.p.) injection of 4 or 20 mg/kg polyriboinosinic-polyribocytidylic acid [poly (I:C)] at 12.5 dpc. Controls were injected with an equal volume of saline (0.01 ml/g body weight). Gray bars: ACTH concentration, open circles: LIF concentration. *, *p* < 0.05 vs. control in FS. #, *p* < 0.05 vs. control in CSF. Number of dams = 3 or 4 in each group. *Error bars*, SEM.

We measured the concentrations of ACTH in FS and LIF in fetal CSF at 3 h after the injection of poly (I:C) at 12.5 dpc. The concentrations of ACTH in FS and LIF in fetal CSF significantly increased in the Poly 4 group (Fig 4A). In contrast, in the Poly 20 group, the concentration of ACTH in FS and LIF in fetal CSF significantly decreased compared to those in the control group (Fig 4A). In both of the Poly 4 and Poly 20 groups, concentrations of ACTH in FS and LIF in CSF significantly decreased compared to the control levels at 24 h after the MIA (at 13.5 dpc) (Fig 4B). The concentration of LIF in FS showed similar pattern that observed in fetal CSF (data not shown). It was also shown that concentration of ACTH in FS correlated well with that of LIF in fetal CSF (Fig 4C). Next, we examined whether there was a relationship between the concentrations of IL-6 in MS and ACTH in FS as well as between the concentrations of LIF in MS and ACTH in FS. The concentration of IL-6 in MS was not significantly correlated with that of ACTH in FS (S2A Fig, *r* = 0.14, p = 0.64). In contrast, the concentration of LIF in MS was significantly correlated with that of ACTH in FS (S2B Fig, *r* = 0.93, p = 0.02). These results demonstrate that fetal ACTH and LIF, constituting the LIF–ACTH–LIF signal relay, are suppressed by 24 h after the MIA. Interleukin-6 in the fetal CSF was not detected with ELISA in the present study.

### Alteration of gp130 target gene expression and JAK2/STAT3 signaling in the placenta

**Fig 5:**
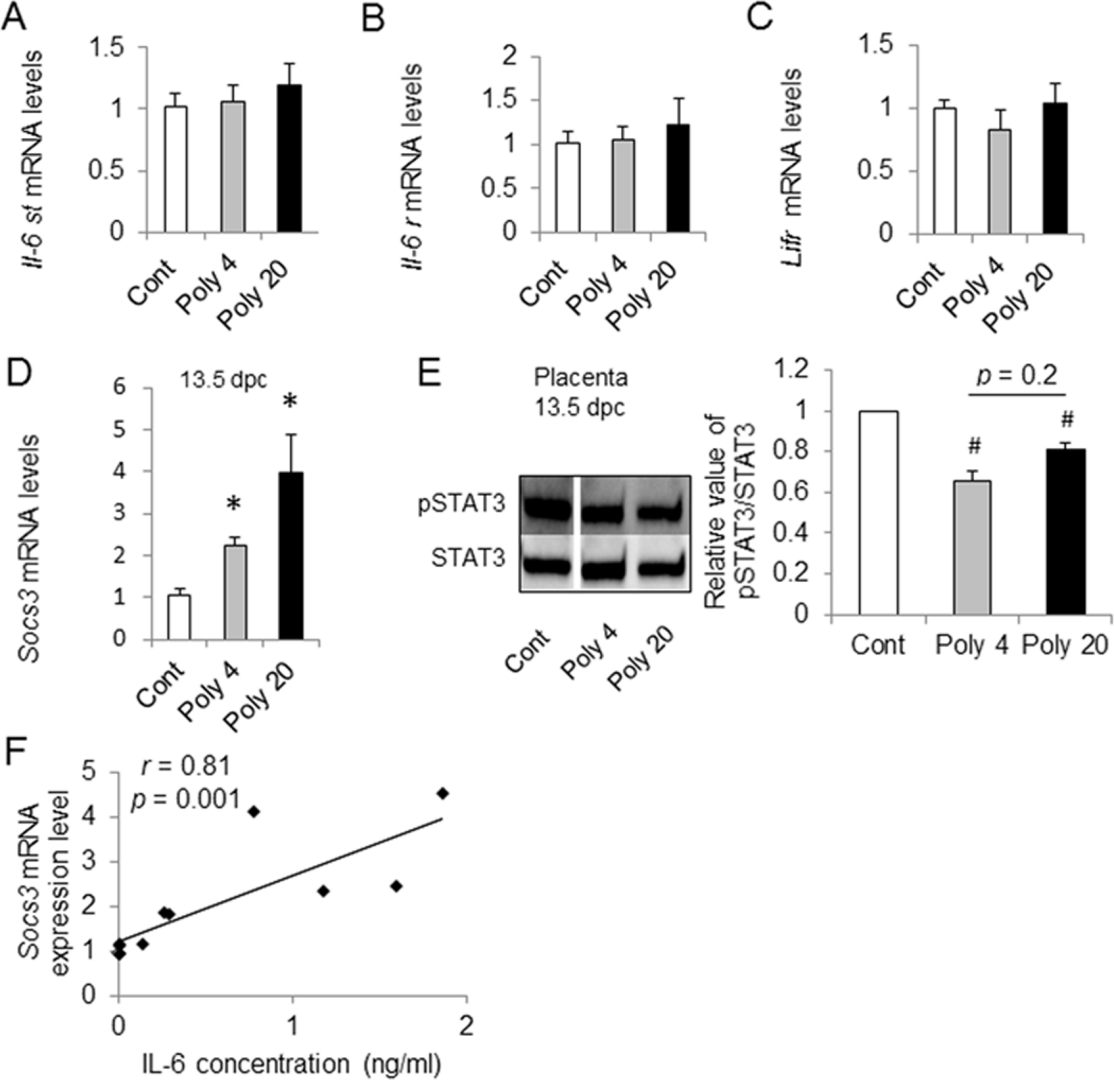
Maternal immune activation (MIA) induced changes of placental gp130 related cytokines gene expression and JAK/STAT3 signaling. (A) Expression of *Il-6st*. (B) Expression of *Il-6r*. (C) Expression of *Lifr*. (D) Expression of suppressor of cytokine signaling 3 (*Socs3*) at 13.5 days post-coitum (dpc). (E) Analysis of STAT3 phosphorylation at 13.5 dpc. (F) Correlation of suppressor of cytokine signaling 3 (*Socs3*) mRNA expression and maternal interleukin-6 (IL-6). Placental *Socs3* expression was upregulated with the polyriboinosinic-polyribocytidylic acid [poly (I:C)] dosage. polyriboinosinic-polyribocytidylic acid [poly (I:C)] at 12.5 dpc, placenta were analysed 3 h after the injection (A, B, C). Expression of *Socs3* and the activation of JAK2/STAT3 signaling were analysed 24 h after the injection (D and E). Controls, Poly 4 and Poly 20 were blotted in the same membrane (E). Maternal IL-6 in control was not detected and is represented as Zero in the Fig (F). Controls were injected with an equal volume of saline (0.01 ml/g body weight). #, *p* < 0.01. *, *p* < 0.05. Number of dams = 3 or 4 in each group, two foetuses were collected as a pooled sample from each litter. *Error bars*, SEM.

To ascertain the signal transduction of LIF in the placenta and the possibility of signal transduction of IL-6 under MIA, we examined the gene expression level of the receptors of LIF and IL-6, and their common signal transducer, gp130, at 3 h after poly (I:C) injection. The expression levels of *Il-6st*, *Il-6r* and *Lifr* did not differ from those of the control (Fig 5A–5C). The level of *Socs3* expression in the placenta exhibited an increase following the dosage of poly (I:C) (Fig 5D). In both of the Poly 4 and Poly 20 groups at 13.5 dpc, the level of *Socs3* expression in placenta was significantly higher than that in the control group. We tested whether the upregulation of *Socs3* expression was linked to the activation of the JAK2/STAT3 signalling pathway. At 24 h after MIA, the relative ratios of phosphorylated STAT3 decreased in both Poly 4 and Poly 20 groups compared with control group (Fig 5E). There were no significant differences in the level of pSTAT3 between the Poly4 and Poly20 groups (p = 0.2). These results show that LIF and IL-6 signals likely regulate the SOCS3 system in the placenta, but IL-6 signals more likely affect the induction of SOC3 because the expression of *Socs3* in the placenta correlates well with IL-6 level in MS (Fig 5F). This finding is supported by the previous report showing the induction of *Socs3* in the placenta with the stimulation of maternal-derived IL-6 [8–10].

## Discussion

In the present study, we show that MIA during mid-gestation suppress the physiological maternal—fetal LIF—ACTH—LIF signal relay and suggest that suppression of the signal relay is mediated by SOCS3 induced by maternal IL-6 in the placenta. Suppression of maternal—fetal LIF signal relay result in reduction of ACTH production from placenta, which lead to a reduction in LIF concentration in fetal CSF, otherwise LIF in fetal CSF reached the peak in mid-gestation. The reduction of fetal CSF LIF in turn impairs the proliferation of the neural stem/progenitor cells, resulting in a smaller cerebral cortex size in late pregnancy. In the rodent models for autism, MIA decreases neurogenesis in the offspring [7, 25–27]. Ischemia or disruption of ventricular surface integrity were so far the possible mechanism proposed to decrease the stem/progenitor cell proliferation in fetal brain following MIA [27, 28]. Here we propose a new molecular mechanism explaining how neural stem/progenitor cell proliferation in fetal brain decreases in MIA.

In contrast to LIF levels, the increase in IL-6 levels in MS is dependent on the poly (I:C) dosage and the expression of *Socs3* in the placenta correlates well with IL-6 level in MS. We therefore consider that IL-6 in MS interferes with the physiological maternal–fetal LIF signal relay and suppresses this signalling by SOCS3 induced by IL-6. *In vitro* studies suggest that IL-6 and LIF can induce the upregulation of *Pomc* expression and bio-active ACTH [14, 29]. But we observed the suppression of ACTH in FS following a reduction in the phosphorylation of STAT3 at 24 h after MIA. In fact, *in vitro* experiments showed SOCS3 to be an intracellular regulator of *Pomc* gene expression and to mediate both *Pomc* promoter activity and ACTH secretion by inhibiting the JAK/STAT3 pathway [30]. Therefore, in the case of MIA, suppression of the maternal–fetal LIF signal relay occurred by 24 h after the MIA.

The maternal–fetal LIF signal relay for neurogenesis had been previously suggested in rats [14]. In the present study on mice, our results demonstrate the existence of a maternal LIF–ACTH–fetal LIF signal relay at 13.5 dpc by presenting exogenous LIF reactivity. MIA suppresses this maternal–fetal LIF signal relay that plays an important role in neurogenesis. Reduced concentration of LIF in fetal CSF may seriously impair fetal brain development, eventually leading to neuropsychiatric disorders. LIF in fetal CSF plays a role in the production of the neural stem/progenitor cells pool. In mice, LIF injection into the fetal cerebral ventricle at 14.5 dpc leads to a thickened cerebral cortex compared with control at 18.5 dpc owing to the over production of neural stem/progenitor cell [11]. Mice lacking gp130, required for LIF signal transduction, show a decreased neural stem/progenitor cell proliferation in the developing cerebrum and the hypoplastic cortical plate [11]. These *in vivo* studies support the critical role played by LIF in fetal CSF during neurogenesis at mid-gestation, and the levels of LIF may determine the size of the cerebral cortex.

The transformation of neural stem/progenitor cells from proliferation to differentiation during brain development is thought to be an important determinant of brain size [31]. During the cerebral cortical development, the symmetrical division of stem/progenitor cells (causing self-renewal) is shifted to asymmetric division of stem/progenitor cells, generating neurons and neuron-producing progenitor cells [32]. These steps of neurogenesis are thought to play an important role in radial and tangential expansion of the cerebral cortex [33]. The time course of this step corresponds to the chronological change of LIF levels in the CSF that showed a peak at 13 and 14 dpc [12], which was suppressed in the fetal brain under the MIA. Previous clinical research reported that LIF gene was associated with schizophrenia [34], this present work may provide a new insight the relationship between MIA and schizophrenia as well as autism in terms of fetal brain development.

Epidemiological studies have shown that adult cardiac and metabolic disorders occur due to fetal origins of early programming of adult disorders [35–37]. Nowadays, this hypothesis leads to the concept of Developmental Origins of Health and Disease; DOHaD [38]. There is significant evidence that maternal virus infection causes the abnormal response against stress, cognitive disturbance and even neuropsychiatric disorders such as schizophrenia and autism in the offsprings of mice and human [39–42]. Moreover, the placental response to a perturbation in the maternal environment is increasingly recognized as having a key role in programming the fetus to develop a disease in adulthood [43, 44].

## Conclusions

We propose a novel mechanism through which MIA suppresses the maternal–fetal LIF signal relay via the placenta, causing the poor development of the fetal brain and increase the risk for neuropsychiatric disorders. By proposing a mechanism linking MIA to neuropsychiatric disorders, this study provides new clues to prevent and treat neuropsychiatric disorders at a prenatal stage.

## Supporting Information

S1 Fig Summary of crown-rump length (CRL) used in this study. CRL were calculated using ImageJ software. There were no significant differences in CRLs among the groups. Cont: control, Poly 4: poly (I:C) 4 mg/kg, Poly 20: poly (I:C) 20 mg/kg, LIF: mouse recombinanat LIF 5 μg/kg. *, *p* < 0.05. Number of dams = 3 or 4 in each group at 12.5 dpc and 14.5 dpc. *Error bars*, SEM.
(TIF)

S2 Fig Correlation of ACTH level in FS with IL-6 level in MS and with LIF level in MS. (A) Correlation of IL-6 level in MS and ACTH level in FS. (B) Correlation of LIF level in MS and ACTH level in FS. Maternal serum and fetal serum were analysed at 3 h after MIA at 12.5 days post-coitum (dpc). MIA was induced by an intraperitoneal (i.p.) injection of either 4 or 20 mg/kg polyriboinosinic-polyribocytidylic acid [poly (I:C)]. ontrols were injected with an equal volume of saline (0.01 ml/g body weight). Number of dams = 3 or 4 in each group.
(TIF)

S1 Table Parameters for stereological analysis. The estimation of volume and total cell number of the cerebral cortex at 18.5 days post-coitum (dpc) was performed using nonbiased stereological method using Stereo Investigator (version 10; Micro BrightField, Williston, VT).
(DOCX)
