# Supplementary figures and images for: The Suppression of Maternal–Fetal Leukemia Inhibitory Factor Signal Relay Pathway by Maternal Immune Activation Impairs Brain Development in Mice
Source: PLoS One. 2015 Jun 4;10(6):e0129011. doi: 10.1371/journal.pone.0129011 (PMC4456156; doi:10.1371/journal.pone.0129011)

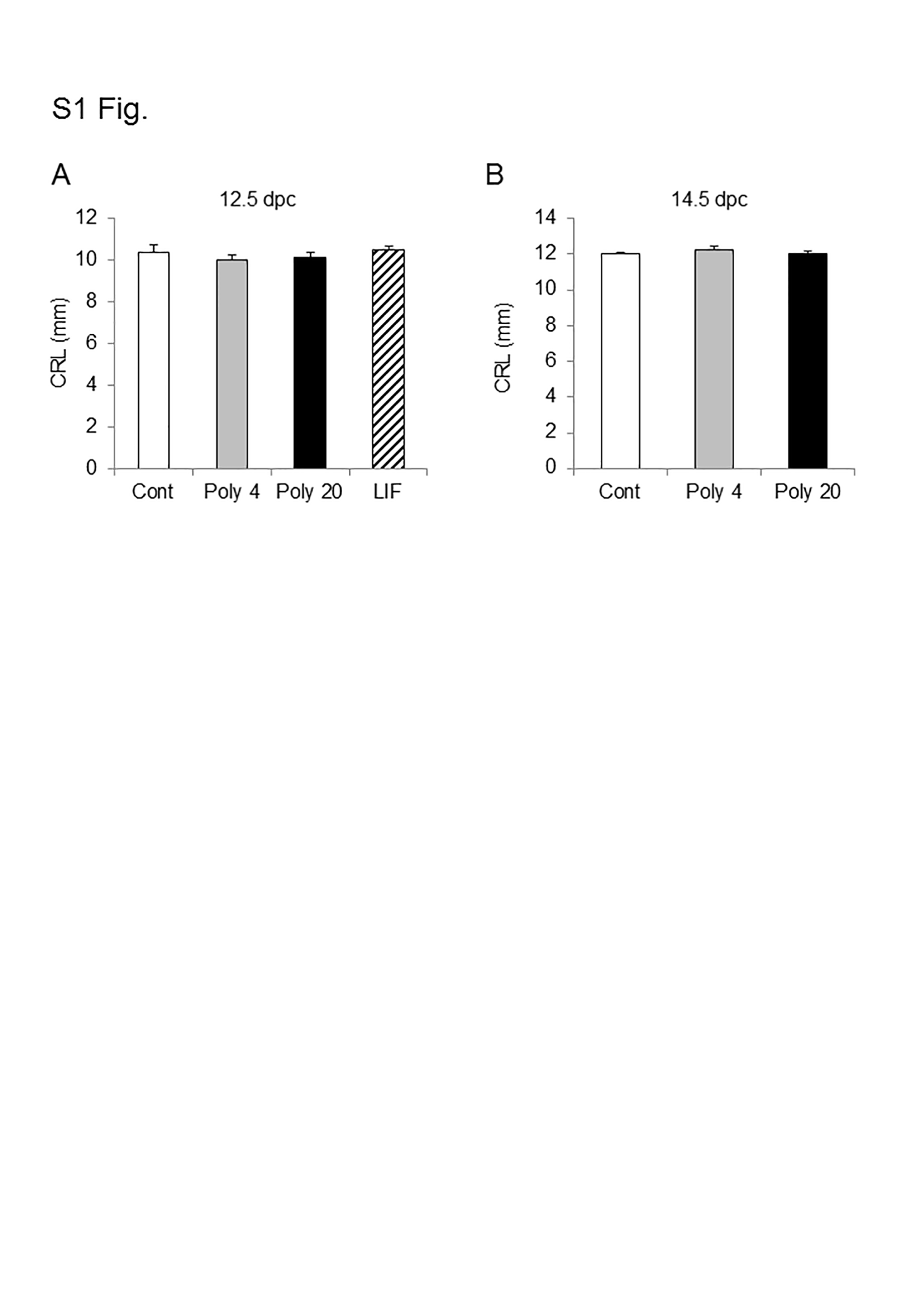

Supplement: S1 Fig — CRL were calculated using ImageJ software. There were no significant differences in CRLs among the groups. Cont: control, Poly 4: poly (I:C) 4 mg/kg, Poly 20: poly (I:C) 20 mg/kg, LIF: mouse recombinanat LIF 5 μg/kg. *, p < 0.05. Number of dams = 3 or 4 in each group at 12.5 dpc and 14.5 dpc. Error bars, SEM. (TIF) [file pone.0129011.s001.tif]

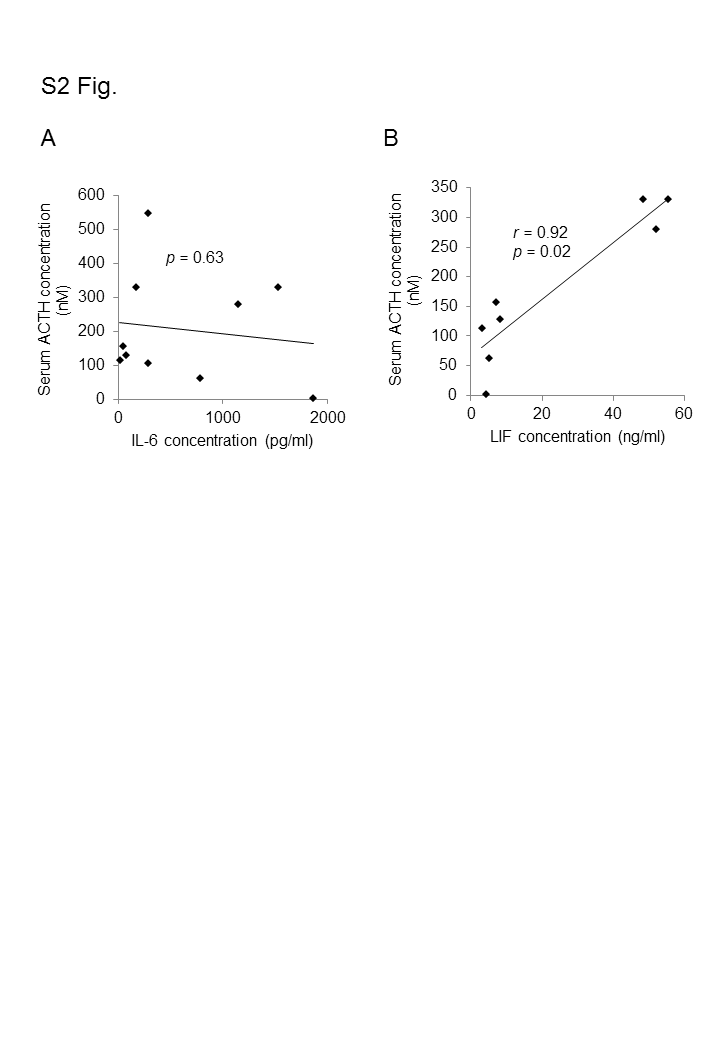

Supplement: S2 Fig — (A) Correlation of IL-6 level in MS and ACTH level in FS. (B) Correlation of LIF level in MS and ACTH level in FS. Maternal serum and fetal serum were analysed at 3 h after MIA at 12.5 days post-coitum (dpc). MIA was induced by an intraperitoneal (i.p.) injection of either 4 or 20 mg/kg polyriboinosinic-polyribocytidylic acid [poly (I:C)]. ontrols were injected with an equal volume of saline (0.01 ml/g body weight). Number of dams = 3 or 4 in each group. (TIF) [file pone.0129011.s002.tif]
