# Supplementary material for: The Suppression of Maternal–Fetal Leukemia Inhibitory Factor Signal Relay Pathway by Maternal Immune Activation Impairs Brain Development in Mice
Source: PLoS One. 2015 Jun 4;10(6):e0129011. doi: 10.1371/journal.pone.0129011 (PMC4456156; doi:10.1371/journal.pone.0129011)
Supplement: S1 Table — The estimation of volume and total cell number of the cerebral cortex at 18.5 days post-coitum (dpc) was performed using nonbiased stereological method using Stereo Investigator (version 10; Micro BrightField, Williston, VT). (DOCX) [file pone.0129011.s003.docx]

S1 Table

| Parameters | Cont | Poly 4 | Poly 20 |
| --- | --- | --- | --- |
| Sections counted | 25.33±0.33 | 25.00±0.58 | 23.00 |
| Number weighted section thickness (µm) | 16.23±0.32 | 16.17±0.32 | 16.00±0.24 |
| Sampling sites | 297.33±15.72 | 201.00±46.03 | 207.33±27.05 |
| Markers counted | 999.33±31.20 | 723.67±77.23 | 729.33±113.50 |
| Counting frame (XY, µm) | 15 × 15 | 15 × 15 | 15 × 15 |
| Grid size (XY, µm) | 200 × 200 | 200 × 200 | 200 × 200 |
| Disector (µm) | 10 | 10 | 10 |
| Coefficient of error (Gunderson's, m=1) | < 0.03 | < 0.04 | < 0.04 |
